# Overcoming Resistance to PARP Inhibitors in BRCA-Mutated Cancers: Mechanisms and Therapeutic Strategies

**DOI:** 10.7150/jca.130206

**Published:** 2026-09-03

**Authors:** Jiahui Guo, Yilei Wang, Jie Li, Zhitao Jiang

**Affiliations:** 1 Department of Pharmacy, Zhangjiagang TCM Hospital, Affiliated to Nanjing University of Chinese Medicine, Zhangjiagang, Jiangsu, 215600, China.; 2 School of Medicine, Nanjing University of Chinese Medicine, Nanjing, Jiangsu, 210023, China.; 3 Department of Pharmacy, Taicang TCM Hospital, Affiliated to Nanjing University of Chinese Medicine, Taicang, Jiangsu, 215400, China.

**Keywords:** PARP inhibitors, BRCA1/2 mutations, synthetic lethality, tumor therapy, PARPi resistance

## Abstract

The tumor suppressor genes BRCA1 and BRCA2 are essential for homologous recombination (HR) repair. Mutations in BRCA1/2 compromise HR repair efficacy, resulting in heightened susceptibility of tumor cells to poly (ADP-ribose) polymerase inhibitors (PARPi) via synthetic lethality. To date, several PARPi have been approved for BRCA-mutated and HR-deficient tumors, but acquired resistance and cumulative toxicity continue to limit their long-term clinical benefit. This narrative review offers an updated mechanistic model of PARPi action and categorizes resistance mechanisms by clinical relevance and evidentiary strength. Importantly, we distinguish between standard combination therapies in the PARPi-naïve setting and interventions explicitly tailored for PARPi-exposed or PARPi-resistant disease. Building on this distinction, we propose a biomarker-guided framework that links specific resistance phenotypes to appropriate specimen types, detection assays, and candidate interventions. Through this evidence-based clinical framework, we aim to clarify the current therapeutic landscape, improve data interpretation, and guide prospective trial design.

## 1. Introduction

DNA damage is generated by endogenous metabolic processes and by exogenous agents, including chemicals, ionizing radiation, and ultraviolet light 1-5. Lesions range from base mismatches and DNA crosslinks to single-strand breaks (SSBs) and double-strand breaks (DSBs) 6. Although an SSB does not immediately disrupt both strands of the DNA duplex, an unrepaired SSB encountered by a replication fork may generate a single-ended or double-ended DSB 7, 8. DSBs are particularly cytotoxic because inaccurate repair can produce deletions, translocations, and other chromosomal abnormalities that compromise genomic stability 5, 9, 10. Major DSB repair pathways include nonhomologous end joining (NHEJ), homologous recombination (HR), single-strand annealing (SSA), and microhomology-mediated end joining (MMEJ) 11-13. NHEJ directly ligates DNA ends throughout the cell cycle and is often error-prone, whereas polymerase theta (Polθ)-dependent MMEJ uses short microhomologies, typically 2-20 nucleotides 13-21. HR is largely restricted to the S and G2 phases, uses a homologous template, usually the sister chromatid, and is therefore a high-fidelity repair pathway 22. BRCA1 promotes repair pathway choice and DNA end resection, whereas 53BP1-dependent end protection favors NHEJ; the balance between these activities is a key determinant of repair outcome 23-25.

BRCA1 and BRCA2 were identified in the 1990s as major tumor suppressor genes, and pathogenic variants in both genes increase the risk of breast, ovarian, and several other cancers 26, 27. Although the two proteins cooperate in HR, their functions are not identical. BRCA1 acts mainly upstream by coordinating checkpoint signaling, promoting DNA-end resection, and directing lesions toward HR during S/G2 28, 29. BRCA2 acts downstream by loading and stabilizing RAD51 on single-stranded DNA (ssDNA), enabling homology search and strand invasion 30, 31. Both proteins also participate in the response to replication stress, but BRCA2 has a particularly direct role in stabilizing RAD51 filaments and protecting nascent DNA at stalled replication forks. These functional differences are relevant to resistance because mechanisms that partly bypass BRCA1 loss may not compensate for the loss of BRCA2-mediated RAD51 loading 32.

PARP1 detects DNA lesions, promotes local chromatin remodeling, and organizes repair through poly(ADP-ribosyl)ation. PARPi inhibit catalytic activity and can stabilize PARP1-DNA complexes, thereby increasing replication-associated damage in HR-deficient cells 33-37. The term BRCAness is more appropriately used for non-BRCA tumors that phenocopy selected features of BRCA deficiency and should not be treated as synonymous with a germline BRCA mutation 38, 39. The resulting synthetic lethal interaction preferentially affects tumor cells that have lost both functional BRCA alleles, whereas most normal tissues in germline mutation carriers retain one wild-type allele and greater HR capacity 40-44. PARPi have produced substantial clinical benefit in several BRCA1/2-mutated cancers, but resistance, toxicity, and differences among tumor types and treatment settings limit their effectiveness 45-47. This review therefore summarizes BRCA1/2 biology, updates the mechanistic basis of PARP inhibition, ranks resistance mechanisms by clinical relevance and evidentiary strength, and evaluates biomarker-guided strategies for PARPi-exposed or PARPi-resistant disease.

### 1.1 Scope and evidence-synthesis approach

This article is a narrative review focused primarily on cancers carrying pathogenic germline or somatic BRCA1/2 alterations. Evidence from non-BRCA HR deficiency, BRCAness, other DNA repair defects, or genotype-independent combinations is included only when it clarifies PARPi mechanism, resistance, biomarker performance, or therapeutic generalizability; these categories are not treated as biologically interchangeable. A focused search of PubMed/MEDLINE and ClinicalTrials.gov was conducted for English-language reports published from January 2005 onward using terms related to PARP inhibition, BRCA1/2, HR, resistance, reversion, replication forks and gaps, transcription-replication conflicts, ctDNA, RAD51 foci, biomarkers, and relevant agents or trials. Official FDA, EMA, and NMPA sources were consulted in the revised evidence synthesis for regulatory-status information. Evidence was interpreted qualitatively as regulatory or randomized clinical evidence, prospective or longitudinal patient evidence, patient-derived translational evidence, or mechanistic cell-line/animal evidence. Because the aim was conceptual and translational synthesis, formal meta-analysis and risk-of-bias scoring were not performed.

## 2. BRCA1/2 and HR Repair

**Figure 1:**
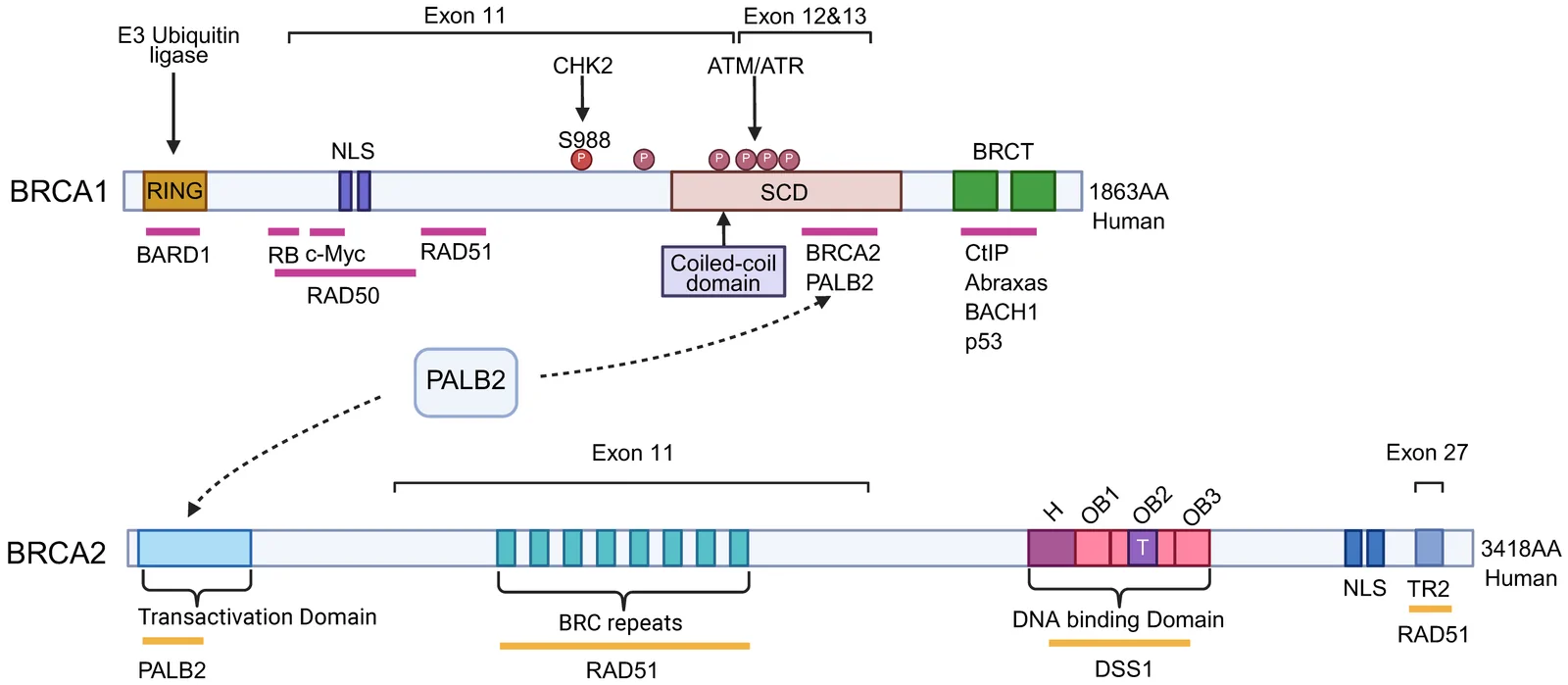
** Main Functional Domains of BRCA1 and BRCA2.** BRCA1 comprises 1,863 amino acids and contains an N-terminal RING domain, two nuclear localization signals (NLSs), a serine cluster domain (SCD), a coiled-coil (CC) domain, and tandem C-terminal BRCT repeats. The RING domain interacts with BARD1; the SCD spans exons 11-13 and contains multiple ATM/ATR-responsive phosphorylation sites; and the BRCT repeats recognize phosphorylated partners including CtIP, Abraxas, and BACH1. BRCA2 comprises 3,418 amino acids. Exon 11 contains eight conserved BRC repeats that mediate RAD51 interactions. The N-terminal region binds PALB2 and maintains the BRCA1-PALB2-BRCA2 axis. The C-terminal region contains nuclear localization signals and an additional RAD51-binding site encoded by exon 27. The DNA-binding domain (DBD) comprises an alpha-helical region, three oligonucleotide-binding (OB) folds, and a tower domain; it interacts with DSS1 to support HR repair.

BRCA1 is located on chromosome 17 and contains 22 coding exons spanning approximately 100 kb of genomic DNA 40. It encodes a 1,863-amino-acid protein of approximately 220 kDa that participates in DNA damage repair, cell-cycle control, and transcription 48-50. BRCA1 contains two nuclear localization signals and three major functional regions: an N-terminal RING domain, a central coiled-coil (CC) domain, and tandem C-terminal BRCT repeats. The RING domain forms an obligate heterodimer with BARD1 and contributes to E3 ubiquitin-ligase activity and damage-site recruitment 51-53. A serine cluster domain within exons 11-13 contains multiple ATM/ATR-responsive phosphorylation sites that help coordinate checkpoint signaling and localization after DNA damage 54. The CC domain binds PALB2 and links BRCA1 to the PALB2-BRCA2-RAD51 axis 55. The tandem BRCT domains, separated by a short linker, recognize phosphorylated partners such as CtIP, Abraxas, and FANCJ/BACH1/BRIP1; pathogenic variants in this region can disrupt substrate recognition, localization, and checkpoint function 56-59 (Figure 1 and Table 1).

BRCA2 is located on chromosome 13, contains 27 coding exons, and encodes a 3,418-amino-acid protein 60. Exon 11 contains eight conserved BRC repeats that interact with RAD51, promote RAD51 loading onto ssDNA, and regulate its association with double-stranded DNA 61-63 (Figure 1). The N-terminal region binds PALB2 and maintains the BRCA1-PALB2-BRCA2 repair axis 54, 64. The C-terminal region contains nuclear localization signals and an additional RAD51-binding site encoded by exon 27; phosphorylation of S3291 by cyclin-dependent kinases regulates this interaction and contributes to replication-fork protection 65, 66. The DNA-binding domain includes an alpha-helical region, three oligonucleotide-binding folds, and a tower domain, allowing BRCA2 to engage both ssDNA and double-stranded DNA. DSS1 stabilizes BRCA2, promotes its nuclear localization, and facilitates RAD51 loading onto RPA-coated ssDNA 67-69. Beyond canonical HR, BRCA2 suppresses post-replicative ssDNA gaps, protects nascent DNA from nuclease-mediated degradation, and contributes to cytokinesis and chromosomal stability 70-73.

HR repairs DSBs and replication-associated lesions by using an intact homologous template 74. BRCA1 and BRCA2 are central regulators of homology-directed repair, and functional disruption of either protein can compromise genome maintenance and therapy response 75, 76. BRCA1 functions upstream in this pathway through interactions with phosphorylated CtIP and the MRE11-RAD50-NBS1 (MRN) complex. These interactions promote 5′ to 3′ DNA end resection and generate the 3′ ssDNA overhang required for RAD51 filament formation 58, 77, 78. CDK-dependent phosphorylation of CtIP facilitates BRCA1 binding and limits RIF1-dependent end protection. In parallel, the BRCA1-BARD1 heterodimer modifies chromatin near DSBs and antagonizes 53BP1, thereby restricting rapid but potentially inaccurate NHEJ and favoring HR 79. This upstream role explains why loss of 53BP1-RIF1-Shieldin can partially restore HR in some BRCA1-deficient models.

BRCA2 acts downstream to assemble and stabilize the RAD51 nucleoprotein filament. After resection, replication protein A (RPA) protects ssDNA from nucleases. PALB2 bridges BRCA1 and BRCA2 at damaged chromatin, after which BRCA2 replaces RPA with RAD51 and stabilizes the resulting RAD51-ssDNA filament 65, 78. RAD51 then searches for a homologous sequence on the sister chromatid and promotes strand invasion and D-loop formation. DSS1 assists this process by weakening RPA-ssDNA interactions and stabilizing the BRCA2-DSS1-RAD51 complex 80. The PALB2-BRCA2-RAD51 axis favors high-fidelity HR and limits mutagenic alternatives such as RAD52-dependent SSA 81, 82. BRCA2 also helps restrain inappropriate end joining and protects stalled replication forks. Therefore, restoration of DNA end resection alone generally cannot compensate for BRCA2 deficiency 83.

## 3. Antitumor Mechanisms of PARPi

ADP-ribosylation is a reversible post-translational modification that uses nicotinamide adenine dinucleotide (NAD+) as the donor of ADP-ribose units 84, 85. Attachment of one unit is termed mono(ADP-ribosyl)ation, whereas polymer formation is termed poly(ADP-ribosyl)ation (PARylation). In humans, these reactions are catalyzed by the PARP/ADP-ribosyltransferase family, whose members regulate cellular stress responses, chromatin structure, DNA repair, and cell death 10, 86, 87. PARP1, PARP2, and PARP3 are DNA-damage-responsive family members, with PARP1 accounting for most damage-induced PAR synthesis and therefore representing the best-characterized therapeutic target 88. PARP2 shares substantial similarity with the PARP1 catalytic domain but lacks the N-terminal zinc-finger architecture. Their partially overlapping functions and the embryonic lethality associated with combined loss emphasize the importance of PAR metabolism in genome maintenance 89.

**Figure 2:**
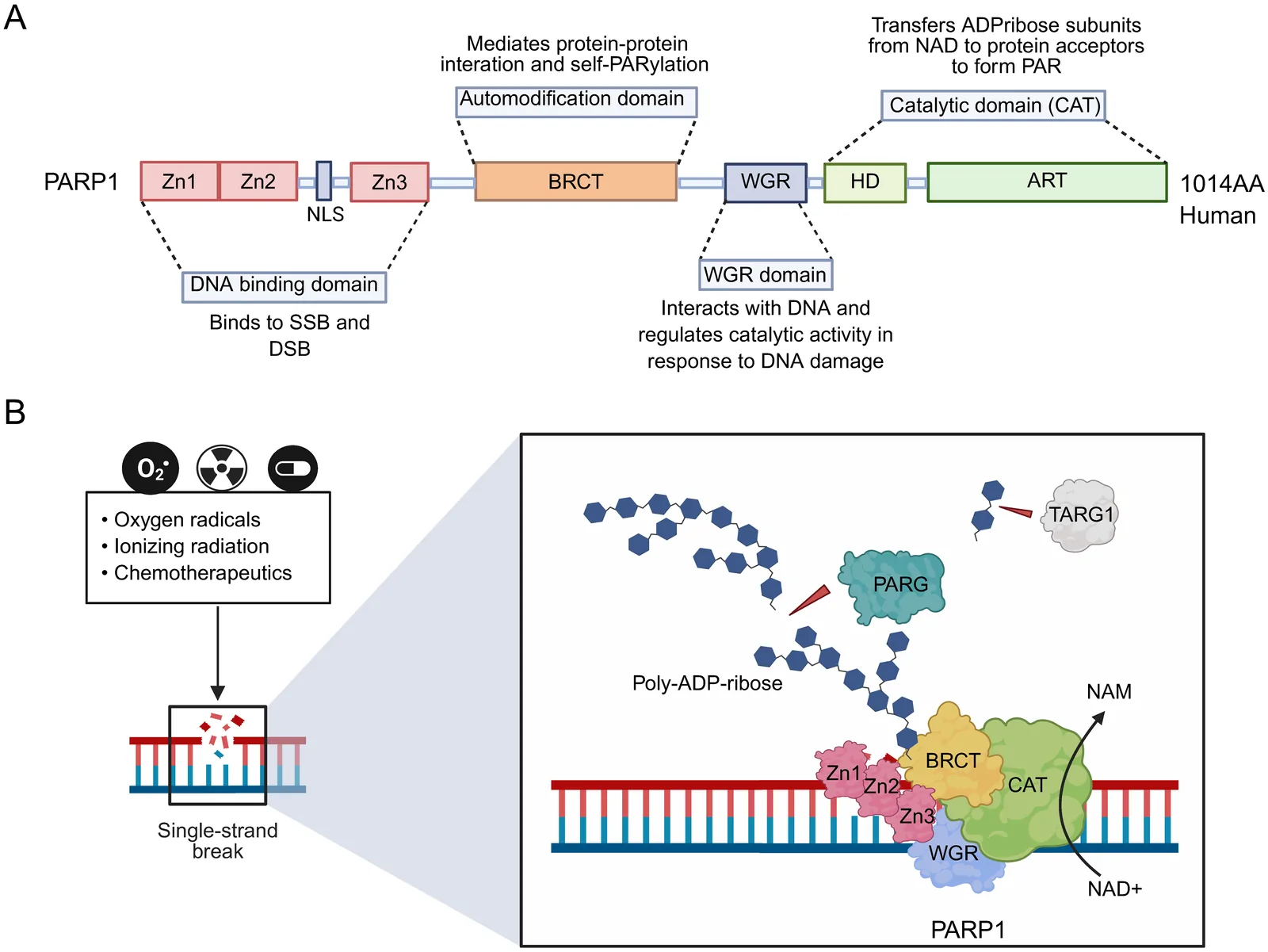
** PARP1 Domains and PAR Metabolism Relevant to PARP Inhibition.** (A) PARP1 contains an N-terminal DNA-binding region with Zn1, Zn2, and Zn3 zinc-finger domains, an NLS between Zn2 and Zn3, a central automodification region containing the BRCT domain, a WGR domain, and a C-terminal catalytic (CAT) domain composed of the helical (HD) and ADP-ribosyltransferase (ART) domains. (B) After binding a DNA nick or break, PARP1 uses NAD+ to synthesize PAR. Extensive auto-PARylation promotes PARP1 release from DNA through electrostatic repulsion. PARG degrades PAR chains, whereas TARG1 removes terminal mono(ADP-ribose), maintaining dynamic PAR turnover.

PARP1 was initially characterized as a factor in SSB repair but is now recognized as a multifunctional regulator of several DNA repair and chromatin-remodeling processes 90. Its N-terminal Zn1 and Zn2 domains recognize DNA discontinuities, whereas Zn3 and the WGR domain transmit the DNA-binding signal to the catalytic region. A nuclear localization signal lies between Zn2 and Zn3, and the central automodification region provides major sites for protein interaction and auto-PARylation. The C-terminal catalytic domain contains a helical domain and an ADP-ribosyltransferase domain that binds NAD+ and synthesizes PAR 91-94 (Figure 2A). DNA binding induces a conformational change that activates catalysis, producing a dense, negatively charged PAR scaffold that promotes chromatin relaxation and the recruitment or organization of repair factors 95-100. Extensive auto-PARylation facilitates PARP1 release from DNA. PARG then hydrolyzes PAR chains, and TARG1 removes terminal mono(ADP-ribose), maintaining a dynamic cycle of PAR synthesis and degradation 101, 102 (Figure 2B).

PARPi exploit the dependence of BRCA1/2-deficient cells on PARP1-mediated responses to replication-associated damage 103. Catalytic inhibition reduces PAR synthesis and impairs repair-factor organization, whereas inhibition of auto-PARylation can prolong the residence of PARP1 at DNA lesions, a process commonly termed PARP1 trapping 18, 104, 105. The traditional model proposes that unrepaired SSBs are converted into DSBs during replication and become lethal when HR is defective. This explanation remains useful but is not sufficient as a universal linear pathway. PARPi can also promote post-replicative ssDNA gaps, abnormal fork remodeling, fork stress, and transcription-replication conflicts. PARP1, TIMELESS, and TIPIN help protect replisomes during such conflicts. In BRCA-deficient cells, unresolved conflicts can generate persistent DNA damage and abnormal mitotic chromatin 106. Thus, catalytic inhibition, trapping, ssDNA-gap accumulation, replication stress, and transcription-replication conflicts should be viewed as parallel and interacting sources of cytotoxicity whose relative contributions vary with the drug, BRCA genotype, and cellular context.

PARPi have demonstrated clinical activity in selected ovarian, breast, pancreatic, and prostate cancers, but regulatory indications differ by jurisdiction, biomarker requirement, and treatment setting and continue to evolve 107-114. Olaparib, rucaparib, niraparib, talazoparib, fuzuloparib, and pamiparib illustrate the diversity of approved agents and regional labels; fixed statements about a single worldwide number of approved PARPi should therefore be avoided. Olaparib has generated pivotal evidence in BRCA-mutated ovarian, breast, pancreatic, and prostate cancers 108-110, whereas other agents have setting-specific roles, including ovarian maintenance, BRCA-mutated breast cancer, and selected regional indications 111-114. Importantly, benefit in a first-line or maintenance trial does not demonstrate reversal of established PARPi resistance. Table 2 is retained as a historical overview rather than a substitute for current FDA, EMA, NMPA, or other local prescribing information. Accordingly, approved indications and PARPi-naïve treatment strategies should be distinguished from the investigational resistance-specific approaches discussed in Section 5.

The synthetic lethal activity of this drug class is driven principally by PARP1, whereas inhibition of PARP2 and other PARP family members may contribute to hematologic and other toxicities. This has encouraged the development of more selective PARP1 inhibitors. Saruparib (AZD5305) is a highly selective PARP1 inhibitor and trapper that has shown favorable target selectivity, tolerability, and preliminary activity in early clinical development 115, 116. However, saruparib remains investigational. It should not be described as an approved treatment or as a proven means of overcoming a specific resistance mechanism, although its wider therapeutic index may support future combination strategies and evaluation in PARPi-exposed populations.

## 4. Mechanisms of PARPi Resistance

**Figure 3:**
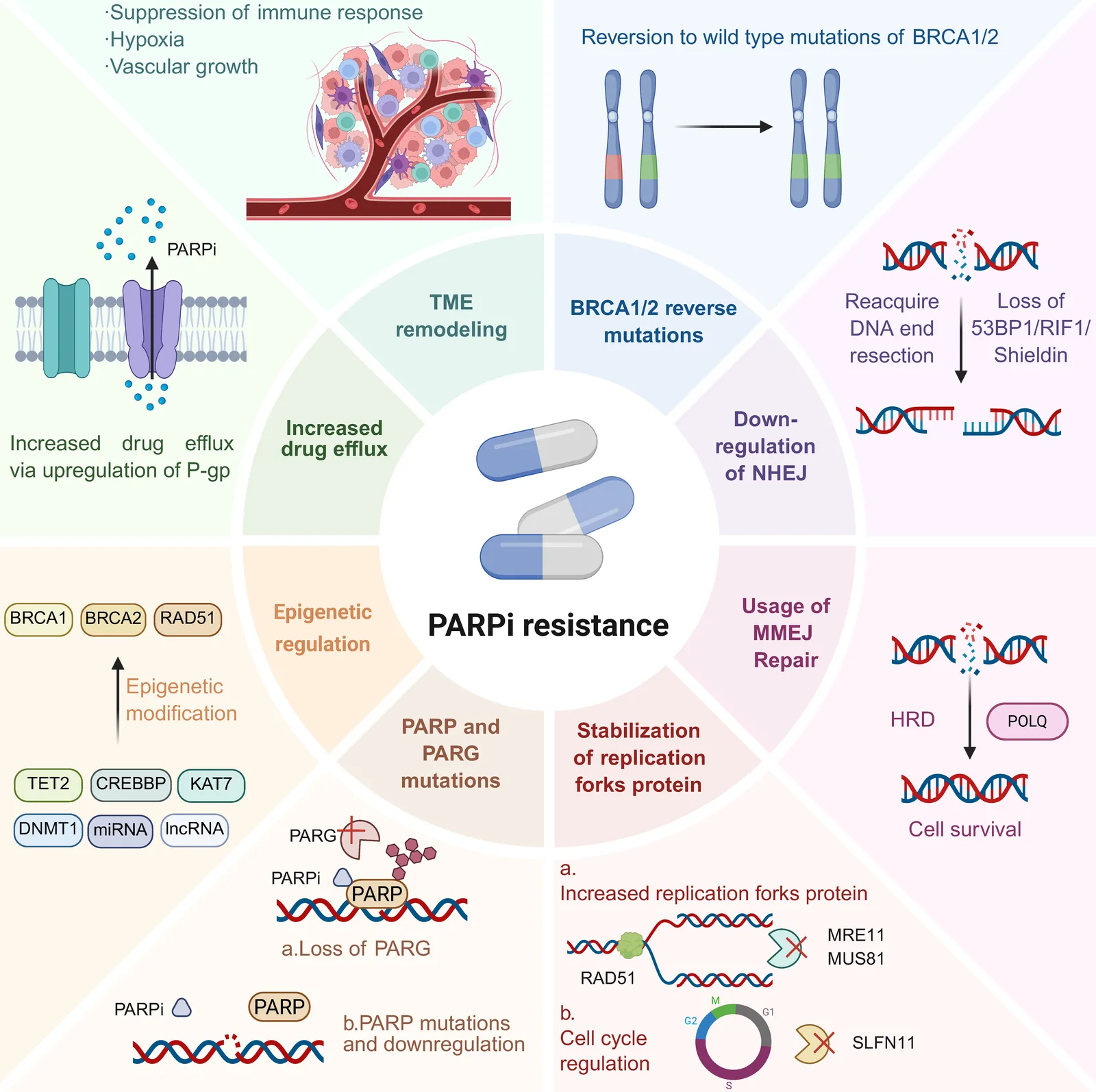
** Mechanisms of PARPi Resistance.** Major mechanisms include BRCA1/2 reversion and restoration of HR, loss of end protection, replication-fork stabilization, altered PARP/PARG biology, increased drug efflux, epigenetic regulation, and tumor microenvironment remodeling. BRCA1/2 reversion and HR restoration have the strongest patient-level support; fork protection, altered PARP/PARG biology, and drug efflux are translationally supported but context-dependent and epigenetic or microenvironmental remodeling remains predominantly preclinical.

PARPi resistance may be intrinsic, adaptive, or acquired. Intrinsic resistance refers to the absence of meaningful initial benefit despite adequate drug exposure, whereas acquired resistance follows a period of clinical response or disease control 117. A fixed duration threshold is not biologically universal and should not replace trial-specific definitions. Adaptive drug tolerance describes a reversible or semi-stable state in which cells survive treatment without an immediately identifiable genetic driver. The proposed progression from a tolerant persister state to stable genetic escape is a useful evolutionary model, but it is not obligatory: tumors may contain pre-existing resistant clones, acquire several mechanisms simultaneously, retain non-genetic resistance, or develop different mechanisms at separate metastatic sites 118, 119. For clinical interpretation, the mechanisms discussed below can be viewed in a practical hierarchy. BRCA1/2 reversion and restoration of HR have the strongest patient-level support; fork protection, altered PARP/PARG biology, and drug efflux are translationally supported but context-dependent; and epigenetic or microenvironmental remodeling remains predominantly preclinical (Figure 3).

The relative contribution of each resistance pathway varies with tumor type, disease stage, prior platinum and PARPi exposure, hormonal signaling, and the timing and method of sampling 120-124. BRCA reversion mutations are well documented in ovarian, breast, pancreatic, and prostate cancers, but their apparent prevalence is cohort-specific. Advanced and heavily pretreated tumors often contain greater clonal diversity and may harbor several concurrent mechanisms 122. Androgen receptor signaling can intersect with DNA-repair programs in prostate cancer, whereas estrogen receptor signaling may influence BRCA and HR-gene expression in breast and ovarian cancers 123, 124. These observations support a tumor- and context-specific interpretation rather than a universal sequence of resistance events.

### 4.1 Restoration of HR Repair

Restoration of BRCA function and HR is the best-established acquired resistance mechanism in BRCA1/2-mutated cancers. Secondary substitutions, insertions, deletions, or structural events may restore the BRCA1/2 open reading frame or generate a partially functional protein, thereby reducing synthetic lethality under platinum or PARPi selection 125, 126. Reversion mutations have been observed after treatment in multiple BRCA-associated tumor types and may arise in several convergent subclones. Longitudinal circulating tumor DNA (ctDNA) can detect some reversion clones before or at radiographic progression and can capture spatial heterogeneity that a single biopsy may miss 118. Nevertheless, a negative plasma result does not exclude resistance because tumor shedding and assay sensitivity vary. Tissue and plasma results should therefore be interpreted together when feasible, and the detection of a reversion does not yet mandate a standardized subsequent therapy. BRCA1 hypomorphic splice isoforms, including Δ11 or Δ11q forms, and HSP90-mediated stabilization of mutant BRCA1 can also restore sufficient function without a full canonical reversion.

RAD51 is central to homology search, strand invasion, and HR restoration. Downregulation of early mitotic inhibitor 1 (EMI1), a component of the ubiquitin-ligase machinery that regulates RAD51 turnover, can increase RAD51 abundance and confer PARPi resistance in BRCA1-deficient models 127. TOPBP1-dependent phosphorylation of RAD51 at serine 14 also influences the replication-stress response, and loss of TOPBP1 can increase PARPi sensitivity 128. More broadly, restoration of RAD51 nuclear foci provides a functional indication that HR competence has returned, regardless of whether the underlying event is a BRCA reversion, altered splicing, increased RAD51 expression, or another pathway change. RAD51-foci assays are therefore promising translational biomarkers, although preanalytical conditions, scoring thresholds, and interlaboratory standardization remain important limitations.

In BRCA1-deficient tumors, loss of end-protection factors can partially restore HR by permitting DNA end resection. Reduced activity of 53BP1, RIF1, Artemis, REV7/Shieldin, or the CST complex can therefore decrease PARPi sensitivity 129-131. This escape route is more biologically plausible in BRCA1 deficiency than in BRCA2 deficiency, because end resection cannot by itself replace the BRCA2-dependent loading of RAD51. MicroRNAs such as miR-622 may also shift repair away from NHEJ by reducing Ku expression 132. Polθ-mediated MMEJ is frequently used by HR-deficient tumor cells and can support survival or generate microhomology-associated reversion events 133. However, MMEJ dependence, HR restoration, and BRCA reversion should be distinguished mechanistically rather than presented as a single equivalent process.

### 4.2 Stabilization of Replication Forks

Replication-fork protection can reduce PARPi sensitivity without fully restoring canonical HR. PARP1, BRCA1, and BRCA2 normally help protect stalled or reversed forks from degradation by nucleases such as MRE11 and EXO1. Loss of PTIP or CHD4 can reduce MRE11 recruitment, while loss of fork-remodeling factors such as SMARCAL1, ZRANB3, or HLTF can limit nuclease-dependent degradation of nascent DNA 134, 135. Alterations involving SLFN11, EZH2-MUS81 signaling, DYNLL1, or RADX can also modify replication-stress tolerance and PARPi response 136-138. These mechanisms are strongly supported in cell lines and patient-derived models, but standardized clinical assays and prospective evidence remain limited. Fork protection should therefore be regarded as a translationally supported, context-dependent mechanism rather than as clinically equivalent to BRCA reversion.

### 4.3 Increased Drug Efflux

Drug efflux lowers the intracellular concentration of anticancer agents and is a recognized mechanism of multidrug resistance. Amplification, promoter rearrangement, or overexpression of ABCB1/MDR1 can increase expression of the membrane transporter P-glycoprotein, which uses ATP hydrolysis to export selected drugs and reduce intracellular exposure 139. ABCB1-mediated resistance has been described in experimental models and some clinical samples after chemotherapy or PARPi treatment. Its relevance, however, depends on whether the specific PARPi is an effective P-glycoprotein substrate. It is therefore inappropriate to assume that all PARPi have identical susceptibility to efflux, and routine clinical testing for this mechanism has not been established.

### 4.4 Decreased PARP Trapping

Alterations in PARP1 structure, activation, or chromatin residence can reduce effective trapping and contribute to resistance. For example, the PARP1 R591C mutation disrupts communication between the WGR and DNA-binding regions, allowing rapid dissociation from damaged DNA and reducing inhibitor-induced trapping 140. Phosphorylation of PARP1 at Y907 by c-MET can enhance catalytic activity and reduce inhibitor binding 141. Conversely, inhibition or loss of the deubiquitinase USP1 increases PARP1 ubiquitination and chromatin retention, thereby enhancing PARPi-induced damage in experimental models 142. These observations provide a mechanistic rationale for USP1- or c-MET-directed combinations, but their prevalence and predictive value in patients with PARPi-resistant disease remain uncertain.

HMGB3 also regulates PARP1 dynamics. HMGB3 is highly expressed in some stem-like and cancer-cell populations and can facilitate PARP1 dissociation from chromatin; its loss or downregulation may increase PARP1 trapping and PARPi sensitivity. Other processes, including TIMELESS-TIPIN-dependent replisome protection and APEX1/KAT6A-associated phase separation, may further influence PARP1 residence at damaged chromatin 143, 144. Most evidence for these mechanisms is preclinical. They should be presented as emerging modifiers of PARP1 trapping rather than as established clinical causes of resistance.

### 4.5 Loss of PARG

PARG reverses PARylation by degrading PAR chains. Loss of PARG or reduced PARG activity can preserve residual PAR signaling despite PARP inhibition, promote PARP1 release from DNA, and maintain limited recruitment of repair factors, thereby reducing PARPi cytotoxicity 145, 146. PARG loss has been identified in resistant experimental models and selected tumor samples, but its clinical frequency and optimal detection method are not yet defined. PARG inhibitors and PARP/PARG combinations are being evaluated preclinically; these approaches remain investigational and should not be interpreted as validated strategies for patients with a detected PARG alteration.

### 4.6 Tumor Microenvironment Remodeling

The tumor microenvironment may modify PARPi response through hypoxia, metabolic stress, stromal signaling, and immune regulation. Moderate hypoxia can reduce reactive oxygen species and attenuate PARPi-associated DNA damage in HR-deficient models, sometimes independently of HIF signaling 147, 148. Hypoxia-activated agents such as tirapazamine have enhanced PARPi activity in experimental systems, but this strategy has not been established clinically. The available evidence therefore supports hypoxia as a plausible, context-dependent contributor rather than a universal resistance mechanism.

PARPi can acutely increase cytosolic DNA and activate cGAS-STING and type I interferon signaling, creating a pro-immunogenic state 149. With prolonged exposure, however, chronic inflammatory signaling may be accompanied by compensatory immunosuppression, including STAT3/IL-34 activation, regulatory T-cell expansion, and cancer-associated fibroblast-derived signals such as CCL2 150. These changes may protect residual tumor cells from immune clearance. Because most data derive from preclinical models or small translational studies, immune remodeling should be classified as an emerging mechanism and not as a dominant explanation for resistance in all BRCA-mutated cancers.

### 4.7 Epigenetic Regulation

Epigenetic changes can alter HR-gene expression and replication-stress tolerance. Promoter methylation of BRCA1, BRCA2, or RAD51C may be associated with an initial HR-deficient state, whereas loss of methylation can restore gene expression and contribute to acquired resistance 151. Genomic scars may nevertheless persist after functional HR has returned, limiting the ability of static HRD scores to represent current drug sensitivity. Changes in DNMT1 activity, TET2-dependent 5-hydroxymethylcytosine, histone acetylation, and chromatin remodeling may also influence replication-fork stability or repair gene transcription 152, 153. Abnormal m6A RNA modification can alter the stability of repair-related transcripts and has been linked to PARPi resistance in experimental models 154. These mechanisms are biologically plausible but are not yet validated as routine treatment-selection biomarkers.

## 5. Strategies for Overcoming PARPi Resistance

**Figure 4:**
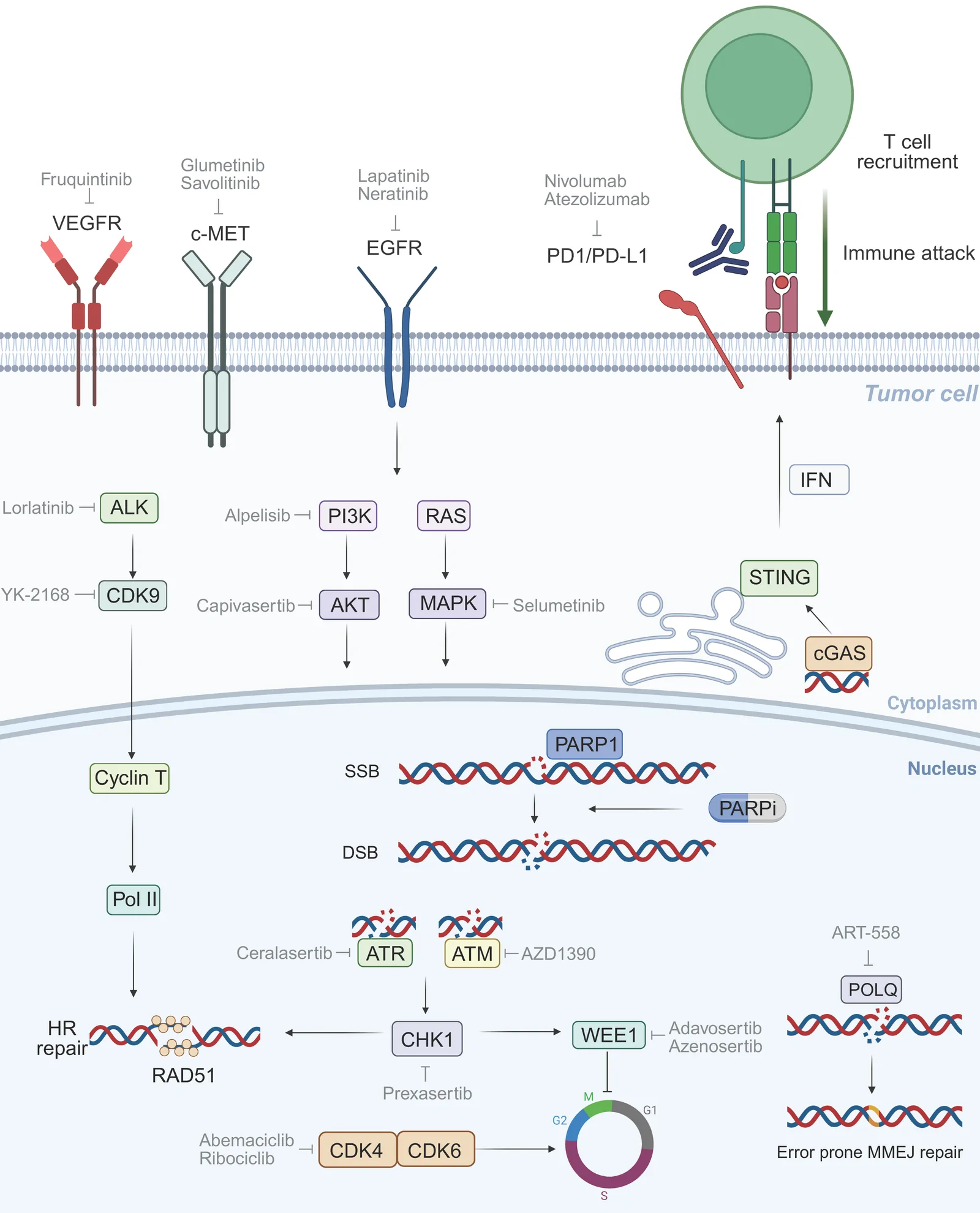
** Mechanisms of Dual-target inhibitors of PARP1.** Investigational approaches include combinations involving immune checkpoint inhibitors, DNA-damage-response inhibitors, Polθ-directed agents, and selected kinase inhibitors. Evidence from approved indications or PARPi-naïve combination settings should not be interpreted as proof of reversal of established PARPi resistance; most strategies shown remain investigational in the resistance-specific setting.

Therapeutic strategies should be interpreted according to both evidence level and treatment setting. A first-line combination may improve initial disease control without reversing an established resistance mechanism; a maintenance trial may enroll platinum-responsive, PARPi-naïve patients; and a preclinical resensitization experiment may demonstrate biological plausibility rather than clinical benefit. The most relevant evidence for overcoming acquired resistance comes from studies that explicitly enrolled patients progressing during or after prior PARPi. At progression, treatment decisions should remain disease-specific and consider platinum sensitivity, prior therapy, toxicity, pace of disease, and access to clinical trials. Biomarker-directed treatment is attractive, but there is limited prospective evidence that biomarker-matched interventions improve clinical outcomes. The sections below retain the original therapeutic categories while clearly distinguishing resistance-specific evidence from broader combination rationale (Figure 4).

### 5.1 Identifying Novel Predictive Biomarkers

Biomarker assessment should distinguish baseline prediction of PARPi benefit from reassessment after progression. At baseline, germline and tumor BRCA testing, biallelic status or loss of heterozygosity, tumor type, prior platinum sensitivity, and treatment setting remain central. After progression, the relevant question is which repair and survival functions are active at that time. Plasma ctDNA can be used for serial monitoring and may identify heterogeneous BRCA reversion clones, whereas tissue biopsy provides histologic context and enables RNA, methylation, protein, and functional assays. RAD51 nuclear foci offer a functional readout of HR competence and may distinguish PARPi-sensitive and PARPi-resistant tumors more directly than BRCA genotype alone 155, 156, but assay induction, cell-cycle controls, thresholds, and reproducibility require further standardization. No single test captures every phenotype: sequencing may detect reversion, methylation assays may identify promoter changes, RNA or protein analysis may support ABCB1-mediated efflux, and specialized pharmacodynamic assays may evaluate PAR metabolism. Together, these complementary assays can support reassessment and clinical-trial selection, but they should not be regarded as a validated prescriptive algorithm.

### 5.2 PARPi Combined with Radiotherapy

PARPi can radiosensitize tumor cells by limiting the repair of radiation-induced SSBs, increasing replication-associated lesions, and modifying stress-response pathways such as NF-κB 157. PARP1/2-deficient cells are more sensitive to ionizing radiation, and low-dose olaparib has enhanced radiosensitization in HR-deficient experimental models 158. Nevertheless, overlapping hematologic and tissue-specific toxicities may constrain dosing, and most studies have not been designed specifically for patients with established PARPi resistance. PARPi-radiotherapy combinations therefore remain investigational as strategies for overcoming resistance.

### 5.3 PARPi Combined with Immune Checkpoint Inhibitors

PARPi-induced DNA damage can activate innate immune signaling, providing a rationale for combination with immune checkpoint inhibitors (ICIs) targeting PD-1/PD-L1 or CTLA-4 159. Early studies have reported activity in several solid tumors. For example, olaparib plus durvalumab, with or without bevacizumab, produced high response rates in selected patients with platinum-sensitive recurrent ovarian cancer, particularly in germline BRCA-mutated disease 160. However, many participants in such studies were not selected for established PARPi resistance, and results across ovarian cancer trials have been inconsistent. Rucaparib plus nivolumab did not consistently outperform PARPi alone, and benefit was not reliably predicted by HRD, BRCA status, or PD-L1 expression 161. PARPi-ICI combinations should therefore not be described as proven resistance-reversal therapy. Better selection based on functional HR status, immune context, and prior PARPi exposure is required.

### 5.4 PARPi Combined with DNA Damage Response Inhibitors

ATR, ATM, CHK1, and WEE1 coordinate cell-cycle checkpoints and replication stress. Inhibiting these pathways can prevent arrest and repair, increase ssDNA and fork instability, reduce RAD51-mediated recovery, and force damaged cells into replication catastrophe or premature mitosis 162. These effects provide a strong rationale for combination with PARPi, particularly when resistant tumors remain dependent on replication-stress checkpoints. Preclinical models and early clinical studies in PARPi-exposed ovarian cancer have shown signals of resensitization, including in BRCA2-mutated disease, but the available cohorts are generally small and heterogeneous. Accordingly, ATR/CHK1-directed combinations should be considered promising but investigational rather than established standards for PARPi resistance.

WEE1 regulates the G2/M checkpoint; its inhibition promotes mitotic entry before DNA repair is complete, leading to genomic instability and mitotic catastrophe 163, 164. WEE1 inhibitors have shown activity in selected platinum-resistant ovarian cancers, including tumors with CCNE1 amplification or TP53 alterations, either alone or with chemotherapy 165, 166. However, hematologic and gastrointestinal toxicities are substantial, particularly when WEE1 inhibition is combined with PARPi or cytotoxic agents. Current development therefore emphasizes dose optimization, intermittent or sequential schedules, and biomarker-enriched populations. WEE1-directed treatment remains investigational in PARPi-resistant disease.

Polθ, encoded by POLQ, is a key enzyme in theta-mediated end joining and is frequently required for the survival of HR-deficient cells. Genetic or pharmacologic Polθ inhibition can produce synthetic lethality and resensitize PARPi-resistant models 167-169. Compounds such as novobiocin, ART558, and related agents have generated encouraging preclinical results, while clinical development remains early. No Polθ inhibitor has yet demonstrated efficacy in a prospectively defined PARPi-resistant population, and the relationship between POLQ dependence, BRCA reversion, and restored HR requires careful biomarker assessment.

### 5.5 PARPi Combined with Protein Kinase Inhibitors

Abnormal kinase signaling can influence HR-gene expression, replication stress, angiogenesis, and PARP1 activity. These observations have motivated combination strategies and dual-target molecules that inhibit PARP1 together with EGFR, CDK4/6, VEGFR, or other kinases 170, 171. EGFR and downstream RAS-MAPK or PI3K-AKT signaling can alter proliferation and DNA repair dependence, while kinase-mediated phosphorylation of PARP1 may reduce inhibitor binding 172. Antiangiogenic approaches may increase hypoxia and replication stress, and CDK4/6 inhibition can reduce the expression of HR proteins in some models, creating a transient HR-deficient phenotype 173. These concepts are mechanistically attractive, but most dual PARP-kinase compounds and many broad kinase combinations remain preclinical or early-phase. Evidence from BRCA wild-type or PARPi-naïve models should not be interpreted as proof that these strategies reverse acquired resistance.

Receptor tyrosine kinases provide additional examples of context-dependent resistance biology. c-MET-mediated phosphorylation of PARP1 at Y907 can enhance PARP1 activity and reduce PARPi binding, and inhibition of c-MET or EGFR has restored sensitivity in experimental systems and selected tumor specimens 141. ALK signaling may increase HR-gene transcription through CDK9 and has been linked to PARPi or platinum resistance. ALK inhibitors combined with olaparib have produced tumor regression in cell-line-derived and patient-derived xenograft models 174, 175. These findings support clinical investigation but remain predominantly preclinical. Regulatory proteins such as HPF1, YB-1, Sam68, BANF1, and TRIP12 may also alter PARP1 activity, stability, or PARylation 33. Their value as patient-selection biomarkers has not been established.

## 6. Conclusions and Perspectives

PARPi have transformed the treatment of selected BRCA1/2-mutated and HR-deficient cancers by exploiting synthetic lethality. Their cytotoxicity is now understood as a nonlinear interaction among catalytic inhibition, PARP1 trapping, post-replicative ssDNA gaps, replication stress, and transcription-replication conflicts rather than as a single SSB-to-DSB pathway. Resistance is similarly heterogeneous. BRCA1/2 reversion and restoration of HR have the strongest clinical support, whereas fork protection, altered PARP/PARG biology, and drug efflux are context-dependent and less readily measured in routine practice. Epigenetic and microenvironmental adaptations remain important research areas but are supported mainly by preclinical evidence. Future studies should combine serial ctDNA, tissue-based molecular and functional assays, and clearly defined clinical settings to distinguish baseline insensitivity from acquired resistance. Trials should also separate PARPi-naïve combinations from true resistance-specific interventions and prospectively test whether biomarker-matched treatment improves outcomes. Selective PARP1 inhibitors such as saruparib may improve the therapeutic index, but they remain investigational and have not yet been proven to overcome a defined resistance mechanism. A clinically useful framework will therefore require both mechanistic precision and evidence-qualified interpretation.

## Figures and Tables

**Table 1:** Functional Domains of BRCA1 and BRCA2

Gene	Domain	Residue range	Principal functional roles and mechanisms
BRCA1	RING domain	24-65	Coordinates zinc ions and forms an obligate heterodimer with BARD1, supporting E3 ubiquitin-ligase activity, DNA-damage signaling, and recruitment to damaged chromatin 51, 52, 176.
	NLS	501-507 and 607-614	Provide binding interfaces for importin-alpha and support active nuclear import of BRCA1-containing complexes 177, 178.
	Serine Cluster Domain (SCD)	1280-1524	Contains multiple ATM/ATR-responsive phosphorylation sites, including Ser1387 and Ser1524, that coordinate localization and S-phase/G2-M checkpoint signaling after DNA damage 54.
	Coiled-coil (CC) domain	1364-1437	Binds PALB2 and promotes assembly of the BRCA1-PALB2-BRCA2 repair complex 55, 82.
	BRCT Repeat Domains	1650-1863	Tandem phosphoprotein-binding repeats recognize phosphorylated CtIP, Abraxas, and FANCJ and coordinate DNA-end resection and damage signaling 56, 57, 59, 179.
BRCA2	N-terminal Binding Region	21-44	Binds PALB2 and EMSY, linking upstream DNA damage signaling to HR and chromatin-regulatory networks 55, 82, 180.
	BRC Repeats (1 through 8)	~1000-2085	Eight conserved repeats within exon 11 interact with RAD51, promote RAD51 recruitment to ssDNA, and regulate RAD51 binding to double-stranded DNA 67, 70.
	DNA-Binding Domain (DBD)	2478-3185	Oligonucleotide-binding folds engage ssDNA and the tower domain contacts double-stranded DNA; DSS1 stabilizes the domain and supports HR and interstrand-crosslink repair 68, 69.
	C-terminal RAD51-Binding Domain (TR2)	Exon 27 (includes S3291)	Provides a second RAD51 interaction site, stabilizes mature RAD51 filaments, and protects stalled replication forks. CDK-dependent S3291 phosphorylation regulates this activity during the cell cycle 70, 71, 181.
	NLS	C-terminal basic clusters	Direct nuclear import of the large BRCA2 complex; loss of these signals impairs nuclear localization and repair function 182.

**Table 2:** ** Selected Historical Milestones in PARP Biology and PARP Inhibitor Development.** Regulatory milestones are shown for historical context and do not imply efficacy in established PARPi-resistant disease.

Year	Events	Summary
1960s	Discovery of PAR and PARylation	Pierre Chambon and colleagues identified NAD+-dependent PARylation, establishing the foundation for PARP research 183.
1970s-1980s	Discovery of PARG and expansion of PARP1 biology	PARG was identified, and the roles of PARP1 in PAR signaling, chromatin remodeling, metabolism, cell death, and DNA repair were progressively defined 184-186.
1990s	Identification of BRCA1/2 and characterization of PARP family structure	BRCA1/2 were established as tumor-suppressor genes required for HR, while the domains and functions of PARP family members were increasingly characterized 187, 188.
2005	Demonstration of synthetic lethality between PARP inhibition and BRCA deficiency	Independent studies showed that BRCA1/2-deficient cells are selectively sensitive to PARP inhibition, establishing a therapeutic synthetic-lethal strategy 43.
2009	Phase I clinical development of olaparib	Early clinical testing demonstrated antitumor activity with a manageable safety profile, providing proof of concept for PARPi in patients 189.
2014	First regulatory approval of olaparib	The FDA approved olaparib for a BRCA-associated ovarian cancer setting, marking the clinical translation of PARP-BRCA synthetic lethality 108, 109.
2016	Regulatory approval of rucaparib	Rucaparib became another FDA-approved PARPi in ovarian cancer 111.
2017	Regulatory approval of niraparib	Niraparib expanded PARPi maintenance treatment in ovarian cancer 112.
2018	Regulatory approval of talazoparib	Talazoparib was approved for HER2-negative advanced or metastatic breast cancer with germline BRCA mutations 113.
2021	Regional approvals of fuzuloparib and pamiparib	The NMPA approved fuzuloparib and pamiparib in selected BRCA-mutated ovarian-cancer settings 114.
2022	Clinical development of selective PARP1 inhibition	Early clinical results for the selective PARP1 inhibitor AZD5305 supported further evaluation of a potentially wider therapeutic index 116.
2024	Greater recognition of transcription-replication conflicts	Mechanistic studies identified unresolved transcription-replication conflicts as an important source of PARPi-induced damage in HR-deficient cells, complementing rather than universally replacing trapping-based models 190.
